# Prevalence of chlamydia among HIV positive and HIV negative patients in the Vhembe District as detected by real time PCR from urine samples

**DOI:** 10.1186/s13104-016-1887-8

**Published:** 2016-02-16

**Authors:** Tshepo Malesela Mafokwane, Amidou Samie

**Affiliations:** Molecular Parasitology and Opportunistic Infections Program, Department of Microbiology, School of Mathematical and Natural Sciences, University of Venda, Private Bag X5050, Thohoyandou, 0950 South Africa

**Keywords:** STD, HIV/AIDS, Chlamydia, Real time PCR, Urine

## Abstract

**Background:**

Chlamydia is a bacterial infection that has long plagued humanity as the most commonly contracted STD and is caused by *Chlamydia trachomatis*. With the emergence of HIV/AIDS, sexually transmitted diseases have also re-emerged as a grave public health problem, particularly in developing countries. Updated Information about the relative frequencies in developing countries is sparse. This study aims at establishing the relative occurrence of chlamydia using real time PCR technique in the Vhembe District of South Africa where reports on the prevalence of chlamydia are not available.

**Methods:**

A total of 243 Urine samples were collected from patients attending different ARV clinics in the Vhembe District and genomic DNA was purified using blood genomic DNA kit from Sigma-Aldrich. Real-Time PCR protocol targeting the 16S rRNA gene of *C. trachomatis* was used to confirm the presence of chlamydia among these patients. Demographic information as well as clinical data was collected as well.

**Results:**

Of all the participants, 70.4 % were females. The age varied from 19 to 72 years. The overall prevalence of chlamydia was 32.1 %. The prevalence was significantly higher among females (39.2 %) compared to males (15.5 %) patients (P = 0.001) and was highest among pregnant women followed by patients who had reported any allergic reaction. Among the HIV positive patients, the prevalence was higher among those who were not taking ARV (38.1 %) compared to those who were taking them (28.5 %). The age group within which the highest prevalence was found was between 26–45 years.

**Conclusions:**

The present study shows a high prevalence of chlamydia among HIV and AIDS patients in the Vhembe District emphasizing the need to enhance STI control and particularly chlamydia among all young people. The particularly high prevalence of chlamydia among pregnant women is of great concern as this predisposes them to complications, while allergy migh predispose people to chlamydia infections. Further studies are needed in the general population both HIV positive and HIV negative persons to further determine the impact of these infections in the community.

## Background

With the appearance of HIV/AIDS, sexually transmitted diseases (STDs) have re-emerged as a grave public health problem, particularly in developing countries. There is strong evidence showing that the presence of genital ulcer disease and of some non-ulcerative STDs enhances the transmission of HIV [1]. There are more than 20 types of STIs of which genital chlamydia and gonorrhea are the most prevalent throughout the world [2]. Chlamydia is also known as the silent STI due to its lack of symptoms in infected individuals particularly females. Chlamydia infection can lead to serious sequelae such as pelvic inflammatory diseases (PID), tubal factor infertility, ectopic pregnancy and chronic pelvis pain in women [3]. In men chlamydia leads to non gonococcal urethritis (NGU), infection of the epididymis which is called epididymitis or the inflammation of the rectum known as proctitis [3]. Understanding the epidemiology of chlamydia is therefore the first step towards its control.

The prevalence of Chlamydia varies significantly between regions and countries. Studies conducted in Kenya, Zimbabwe, Nigeria and South Africa, have shown that the prevalence of *C. trachomatis* varied between 6 and 56 % in these countries with most studies reporting prevalence of about 18 % [4–7]. In South Africa specifically, most studies were conducted using very specialized group of patients mostly all from one gendre like make only or female only samples [8, 9]. The Vhembe District is an immediate neighbor to the Mopani District where a new serotype of *C. trachomatis* was discovered although no studies have been conducted in the Vhembe District [10]. The primary prevention of chlamydia in the form of health education and secondary prevention through early diagnosis and treatment are important chlamydia control strategies [11]. Also, the improvement in management of STI’s can reduce the incidence of HIV infection in the general population [12]. *C. trachomatis* infection can be effectively cured with antibiotics such as azithromycin or ofloxacin once it is detected, for pregnant women erythromycin or amoxicillin is recommended [13, 14].

Various methodologies can be used to detect *C. trachomatis* [15]. Cell culture is considered as the gold standard in the diagnosis of *C. trachomatis* however the rapid evolution of the DNA amplification techniques (Polymerase chain reaction for example) is leading to the re-evaluations of this standard by the scientific community [16]. Recently a technological innovation that came from PCR namely real time PCR has become more common for clinical diagnostics because it is run in a closed system and it has the ability to generate both qualitative and quantitative results [17]. Their main advantages are the possibility of quantification, along with greater sensitivity, precision and accuracy as well as faster analysis making this technique superior to the other methods [18]. Hence this study aims at establishing the relative occurrence of chlamydia using real time PCR technique in the Vhembe District of South Africa where reports on the prevalence of chlamydia are not available.

## Methods

### Ethical clearance

The study was approved by the University of Venda Health and Ethics Committee and the Limpopo department of health in Polokwane. Ethical clearance was also obtained from the hospitals where the samples were collected. The objectives of the study were clearly explained to the patients as well as their rights to withdraw anytime and confidentiality. Those who agreed to participate in the research were requested to sign a consent form and complete the questionnaire with the help of a researcher in order to obtain socio-demographic information as well as data about previous sexual experience and the human experimentation guidelines were followed.

### Sample collection and processing

Patients attending the HIV clinics in three major hospitals in the Vhembe District including Elim, Tshilidzini and Donald Fraser hospitals as well as students attending the University of Venda HIV clinic for HIV testing and counseling were recruited in the study. All patients presenting at the above mentioned clinics between April and November 2010, were invited to participate in the study. The main reasons for these patients visiting the clinic were generally to get HIV care which include getting their medication, a follow up visit to check for CD4 count etc.… Urine samples were collected from the study participants who had agreed to take part in the study and were labeled with the patient’s code, sex, age and the collection date. The samples were taken to the University of Venda Microbiology laboratory in cooler boxes with ice within 4 h of collection for analysis. Upon arrival, samples were centrifuged and the pellet was kept at −20 °C for further analysis.

### DNA extraction

Genomic DNA was purified from urine samples using the commercially available blood genomic DNA extraction kit from Sigma Aldrich following the instructions from the manufacturer. The extracted DNA was then stored at −20 °C for further analysis.

### Quantitative real-time PCR

Genomic DNA extracted from the urine samples was used in a real-time PCR protocol using the primers previously described targeting the 16S ribosomal RNA of chlamydia with SYBR-Green-490 (Roche Diagnostics) [16]. The reaction was performed in light cycler 480 from Roche Diagnostics. The results were analyzed with a user-defined threshold of 200 PCR Baseline Subtracted Curve-fit Relative Fluorescence Units (CF RFU). The level of positivity of the samples was then indicated by the cycle threshold (Ct) values, which represent the number of cycles necessary for the samples to cross the threshold: the smaller it is, the more DNA is in the samples. All runs included at least two negative controls and one positive control.

### Statistical analysis

The analysis was conducted using the statistical package for social sciences (SPSS) program, version 17.1 with the fisher Chi square test and the difference between two variables was considered significant if the P value was less than 0.05. Different analysis including the student t test, the multivariate analysis correcting for confounders such as HIV status, the age of the participants, and the pregnancy status. A regression analysis was also conducted to evaluate the potential correlation between chlamydia infections and the different symptoms reported by the patients. In preparation of the regression model a univariate analysis was conducted and the variables that had a P value of 0.1 or less, were retained for the construction of the regression model. Of all the variables, gender, pregnancy, fever and abortion were the variables that remained. Taking specific ARVs particulary zidovudine, tenofovir and stavudine was associated with a reduction on the prevalence of chlamydia. Following the selection using the univariate model, the variables were taken out of the model in s stepwise protocol and a linear regression was built taking into consideration all the variable that had some impact on the prevalence so as gender, fever, or abortion. Pregnancy was also an important factor.

## Results

### Distribution of chlamydia in the study population based on demographic characteristics and HIV status

**Fig 1:**
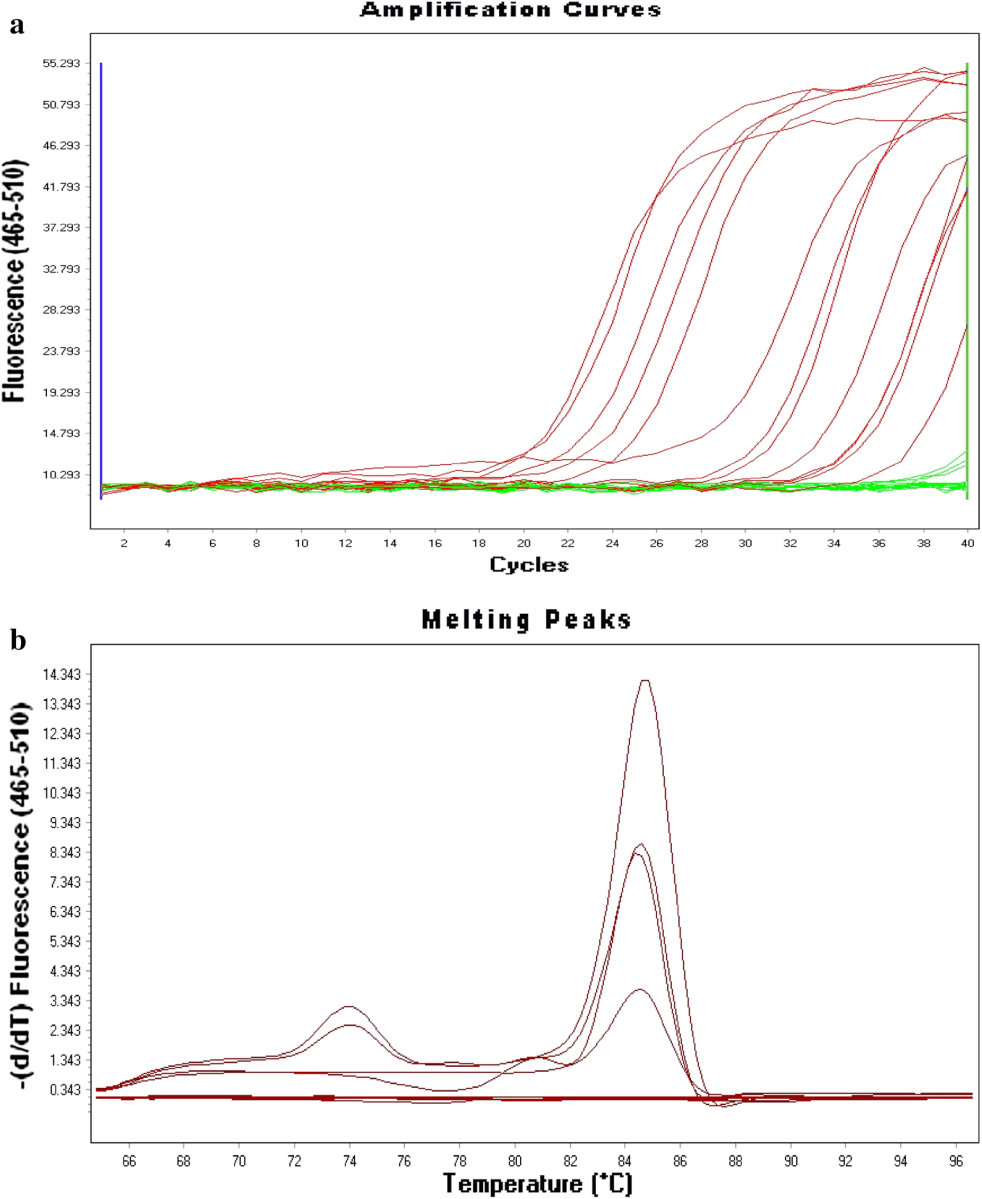
Amplification and melt curves obtained from the Light Cycler 480 II after real time PCR amplification of *Chlamydia trachomatis* DNA from the urine samples obtained from HIV positive and HIV negative patients visiting HIV care centres. **a** shows the amplification curves and** b** shows the melting curve analysis. The minor picks appearing at around 74 °C on the melting curves are probably due to the primer dimers

Following amplification, the amplification and melting curves were generated by the Light Cycler 480 II as showed in Fig. 1. The patients were from different hospitals and a clinic in the University of Venda (Univen). Of the 243 samples collected from as many patients, 78 (32.1 %) were positive for chlamydia. The highest prevalence was found among students in the University with 37.5 %. Female patients had the highest prevalence 39.2 % than males 15.5 % and the difference was statistically significant (P = 0.001). Based on the marital status, widowed patients were the most infected with 46.7 % prevalence and the least infected were the singles with 27.5 %. Most people who were infected were between 25–45 years the least infected were 24 years and younger but the difference was not significant (P = 0.313). The patients’ HIV status did not seem to have an impact on the prevalence of chlamydia in the study sample although only eight HIV negative patients were tested. However, ARV treatment seemed to have a positive impact. In fact among the HIV positive, those who were not taking ARV’s had the highest prevalence 38.1 % than those who were taking them 28.5 %. The results are presented on Table 1. The effects of the different antiretroviral drugs were evaluated on the occurrence of chlamydia. However, none of the drugs used had a significant impact on chlamydia (Table 2). Pregnant women had the highest prevalence of chlamydia compared to men and non-pregnant women and the difference was statistically significant (Table 3).

**Table 1:** Distribution of chlamydia in the study population by origin, age, gender and marital status

Variables	Characteristics	Total (N)	Chlamydia positive N (%)	Statistics (Chi square and P value)	OR (odd ratio), 95 % CI (confidence interval)
Origin	Elim	50	16 (32.0)	χ^2^ = 0.00, P = 0.987	OR = 0.994, 95 % CI 0.0511–1.937
	Tshilidzini	66	18 (27.3)	χ^2^ = 0.968, P = 0.325	OR = 0.731, 95 % CI 0.38–1.35
	Donald Fraser	119	41 (34.50)	χ^2^ = 0.593, P = 0.441	OR = 1.23, 95 % CI 0.721–2.12
	Univen	8	3 (37.5)	χ^2^ = 0.111, P = 0.739	OR = 1.28, 95 % CI 0.29–5.49
Gender	Female	171	67 (39.2)	χ^2^ = 12.888, P = 0.001	OR = 3.514, 95 %CI 1.723–7.16
	Male	71	11 (15.5)		
Marital status	Single	102	28 (27.5)	χ^2^ = 3.99, P = 0.174	OR = 0.681, 95 % CI 0.391–1.187
	Married	90	29 (32.2)	χ^2^ = 0.00, P = 0.998	OR = 0.999, 95 % CI 0.572–1.746
	Divorced	20	7 (35.0)	χ^2^ = 0.077, P = 0.782	OR = 1.145, 95 %CI 0.438–2.994
	Widowed	30	14 (46.7)	χ^2^ = 3.267, P = 0.071	OR = 2.023, 95 % CI 0.932–4.392
Age groups	19–24	5	5 (20)	χ^2^ = 2.112, P = 0.146	OR = 0.476, 95 % CI 0.171–1.320
	25–45	135	48 (35.6)	χ^2^ = 1.015, P = 0.314	OR = 1.332, 95 % CI 0.762–2.327
	Above 45	74	24 (32.4)	χ^2^ = 0.111, P = 0.916	OR = 0.969, 95 % CI 0.538–1.744
HIV Status	HIV negative	8	3 (37.5)	χ^2^ = 0.111, P = 0.739	OR = 0.918, 95 % CI 0.533–1.581
	HIV positive		75 (31.9)		
ARV	Taking ARVs	154	43 (28.5)	χ^2^ = 2.989, P = 0.130	OR = 0.647, 95 % CI 0.368–0.138
	Not taking ARVs	84	32 (38.1)

**Table 2:** Occurrence of Chlamydia in a HIV population in relation to antiretroviral treatment

Treatment	Count	Chlamydia positive (%)	Statistics (χ^2^, P value)	OR (odd ratio and 95 % confidence interval)
Not on ARV	84	32 (38.1)	χ^2^ = 2.365, P = 0.124	OR 0.649, 95 % CI 0.374–1.228
On ARV	133	37 (27.8)		
Zidovudine	12	6 (50)	χ^2^ = 3.158, P = 0.076	OR 2.871, 95 % CI 0.861–9.562
Efavirenz	114	29 (25.4)	χ^2^ = 2.784, P = 0.095	OR 0.426, 95 % CI 0.154–1.184
Lamivudine	129	36 (27.9)	χ^2^ = 0.043, P = 0.836	OR: 0.774, 95 % CI 0.068–8.804
Tenofovir	42	11 (26.2)	χ^2^ = 0.103, P = 0.748	OR 0.873, 95 % CI 0.383–1.993
Nevirapine	15	7 (46.7)	χ^2^ = 2.914, P = 0.088	OR 0.426, 95 % CI 0.154–1.184
Stavudine	81	23 (28.4)	χ^2^ = 0.014, P = 0.906	OR 1.048, 95 % CI 0.479–2.291

**Table 3:** Occurrence of chlamydia in the study population in relation to gender and pregnancy

Population	Count	Chlamydia positive (%)	Statistics (χ^2^, P value)	OR (odd ratio and 95 % confidence interval)
Males	65	10 (15.4)	χ^2^ = 12.88, P = 0.0001	OR 3.514, 95 % CI 1.723–7.165
Non-pregnant females	136	48 (35.3)	χ^2^ = 3.376, P = 0.066	OR 2.095, 95 % CI 0.943–4.658
Pregnant females	30	16 (53.3)	χ^2^ = 7.289, P = 0.007	OR 2.837, 95 % CI 1.302–6.186
Total	231	74 (32)	χ^2^ = 7.183, P = 0.007	OR 2.818, 95 % CI 1.292–6.144

### Impact of education and income status of patients on the prevalence of chlamydia

Based on education level and income of the household head, participants who indicated that they had an income of less than Rands 1000 per month appeared to be slightly higher prevalence of chlamydia (35.4 %) compared to those who had a higher salary (31.3 %). However, the difference was not statistically significant (P = 0.425). Most of the participants who had more than six dependents were more infected (35.4 %) compared to those who had between two and three dependents. Participants who went to secondary school were more infected (35.9 %) than those who went only to primary school (26.6 %). The results are indicated on Table 4.

**Table 4:** Impact of socio-economic status of patients on the prevalence of chlamydia

Variables	Characteristics	Total (N)	Positive N (%)	Statistics
Income range less than 1000	Yes	96	34 (35.4)	χ^2^ = 0.425, P = 0.515, OR = 1.204, 95 % CI 0.68–2.103
	No	131	41 (31.3)	
Number of dependents	1–3 dependents	87	26 (29.9)	χ^2^ = 0.474, P = 0.491, OR = 0.816, 95 % CI 0.457–1.456
	4–6 dependents	98	33 (33.7)	χ^2^ = 0.093, P = 0.760, OR 1.093, 95 % CI 0.622–1.915
	7–13	38	13 (34.2)	χ^2^ = 0.093, P = 0.760, OR = 1.092, 95 % CI 0.522–2.283
Education	Primary school	64	17 (26.6)	χ^2^ = 1.533, P = 0.216, OR = 0.670, 95 % CI 0.355–1.266
	Secondary school	128	46 (35.9)	χ^2^ = 1.259, P = 0.262, OR = 1.367, 95 % CI 0.0.791–2.364
	Tertiary school	20	6 (30.0)	χ^2^ = 0.076, P = 0.782, OR = 0.869, 95 % CI 0.321–2.356
	No education	26	9 (34.6)	χ^2^ = 0.045, P = 0.832, OR = 1.097, 95 % CI 0.465–2.586

### Prevalence of chlamydia in relation to other symptoms experienced by the patients

In relation to the symptoms reported by the patients at the time of sample collection, chlamydia was detected most among patients who reported to have had STI’s with 26.0 %, vision problems with 32.6 %, sores on lips with 31.4 %, rash in genitals with 29.6 % and blind spot with 30.8 % (Table 5). The level of CD4 count as well as the viral load did not seem to affect the prevalence of chlamydia in the study population. Of all the conditions reported by the patients only alergy was significantly statistically associated with high prevalence of chlamydia (Table 5). This association persisted even after the data was segregated by gender. More male patients with allergy had chlamydia compared to males who did not report any allergy while females who reported any type of allergy were significantly more infected with chlamydia.

**Table 5:** Prevalence of chlamydia in relation to the symptoms experienced by the patients

Characteristic	Total	Positive N (%)	Statistics: χ^2^, P value	OR, 95 % CI (odd ratio, 95 % confidence interval)
Pain (general body pain)				
No	127	40 (31.5)	χ^2^ = 0.646, P = 0.412	OR 1.265, 95 % CI 0.712–2.24
Yes	87	32 (36.5)		
Fever				
No	151	54 (35.8)	χ^2^ = 1.029, P = 0.310	OR 0.719, 95 % CI 0.37–1.36
Yes	63	18 (28.6)		
Current chest pain				
No	194	58 (29.9)	χ^2^ = 2.440, P = 0.118	OR 1.675, 95 % CI 0.87–3.21
Yes	48	20 (41.7)		
Allergy (any type of allergy)				
No	180	49 (27.2)	χ^2^ = 6.841, P = 0.009	OR 2.202, 95 % CI 1.21–4.05
Yes	62	28 (45.2)		
Sores in genitals				
No	220	73 (33.2)	χ^2^ = 1.251, P = 0.263	OR 0.559, 95 % CI 0.20–1.56
Yes	23	5 (21.7)		
Sores on lips				
No	208	67 (32.2)	χ^2^ = 0.008, P = 0.927	OR 0.965, 95 % CI 0.44–2.08
Yes	35	11 (31.4)		
Genital warts				
No	212	71 (33.5)	χ^2^ = 1.477, p = 0.224	OR 0.579, 95 % CI 0.23–1.41
Yes	31	7 (22.6)		
Rash in genitals				
No	150	45 (30)	χ^2^ = 0.001, P = 0.969	OR 0.982, 95 % CI 0.40–2.41
Yes	27	8 (29.6)		
Mouth thrush last 3 months				
No	213	67 (31.5)	χ^2^ = 0.490, P = 0.484	OR 1.332, 95 % CI 0.59–2.97
Yes	29	11 (37.9)		
Vision problem				
No	156	49 (31.4)	χ^2^ = 0.034, P = 0.854	OR 1.054, 95 % CI 0.60–1.85
Yes	86	28 (32.6)		
Blurry vision				
No	41	16 (39)	χ^2^ = 1.962, P = 0.165	OR 0.521, 95 % CI 0.20–1.31
Yes	44	11 (25.0)		
Blind spot				
No	72	23 (31.9)	χ^2^ = 0.007, P = 0.933	OR 0.947, 95 % CI 0.26–3.39
Yes	13	4 (30.8)		
STD (general STI symptoms)				
No	191	64 (33.5)	χ^2^ = 1.027, P = 0.311	OR 697, 95 % CI 0.34–1.40
Yes	50	13 (26.0)		
Diabetes				
No	237	78 (32.9)	χ^2^ = 2.908, P = 0.088	OR 0.671, 95 % CI 0.61–0.733
Yes	6	0 (0.0)		
Hypertension				
No	233	74 (31.8)	χ^2^ = 0.299, P = 0.585	OR 1.432, 95 % CI 0.39–5.22
Yes	10	4 (40.0)		
Epilepsy				
No	241	77 (32)	χ^2^ = 0.296, P = 0.586	OR 2.13, 95 % CI 0.130–34.50
Yes	2	1 (50)		
Skin problem				
No	154	50 (32.5)	χ^2^ = 0.082, P = 0.774	OR 0.921, 95 % CI 0.52–1.62
Yes	88	27 (30.7)		
Past skin problem				
No	167	54 (32.3)	χ^2^ = 0.161, P = 0.688	OR 0.887, 95 % CI 0.48–1.60
Yes	74	22 (29.7)		

### Prevalence of chlamydia in relation to the sexual behavior of the participants

Patients were asked in the questionnaires about their sexual behavior, this included the age at first sexual experience, number of partners since first sexual experience, number of partners in the previous year and condom use. Patients who indicated that they had their first sex at an earlier age (between 12 and 15 years) had the highest prevalence (43.8 %). Patients who indicated that they had 1–4 partners since their first sexual experience where more infected (37.2 %) while those who indicated that they had more than 10 partners were the least infected (30.0 %). Patients, who had only one sexual partner in the previous year, were mostly infected (38.6 %) more than those who had 5 or more partners (28.6 %). People who indicated that they use condoms sometimes were more infected (44.7 %) than those who never use condoms (30.8 %) and those who always use it (28.9 %), but the difference was not significant (P = 0.262). The results are recorded in Table 6.

**Table 6:** Prevalence of chlamydia in relation to the sexual behavior of the participants

Variables	Characteristics	Total (N)	Positive N (%)	Statistics	
Age at first sex	12–15 years	16	7 (43.8)	χ^2^ = 0.691, P = 0.406,	OR = 1.544, 95 % CI 0.551–4.327
	16–18 years	108	42 (38.9)	χ^2^ = 2.056, P = 0.152,	OR = 1.511, 95 % CI 0.858–2.662
	19–22 years	70	20 (28.6)	χ^2^ = 3.633, P = 0.045,	OR = 0.681, 95 % CI 0.367–1.264
	23 years and above	16	5 (22)	χ^2^ = 3.633, P = 0.057,	OR = 0.254, 95 % CI 0.056–1.149
No of sex partners since first sex	1–4 sex partners	129	48 (37.2)	χ^2^ = 0.889, P = 0.346,	OR = 1.33, 95 % CI 0.73–2.427
	5–9 sex partners	38	12 (31.6)	χ^2^ = 0711, P = 0.646,	OR = 0.838, 95 % CI 0.395–1.781
	More than 10 partner	40	12 (30.0)	χ^2^ = 0.500, P = 0.480,	OR = 0.764, 95 % CI 0.362–1.612
No of sex partners the previous year	0 partners	64	20 (31.3)	χ^2^ = 0.412, P = 0.521,	OR = 0.815, 95 % CI 0.436–1.524
	1 partner	114	44 (38.6)	χ^2^ = 1.893, P = 0.169,	OR = 1.496, 95 % CI 0.842–2.657
	2–4 partners	27	7 (25.9)	χ^2^ = 0.992, P = 0.319,	OR = 0.631, 95 % CI 0.254–1.571
	5 or more partners	7	2 (28.6)	χ^2^ = 0.110, P = 0.740,	OR = 0.755, 95 % CI 0.143–3.990
Condom use	Always	84	24 (29.8)	χ^2^ = 1.841, P = 0.175	OR = 0.605, 95 % CI 0.292–1.253
	Never	13	4 (30.8)	χ^2^ = 0.070, P = 0.791,	OR = 0.847, 95 % CI 0.246–2.913
	Sometimes	38	17 (44.7)	χ^2^ = 2.677, P = 0.102,	OR = 1.898, 95 % CI 0.876–4.113
Currently abstaining from sex	No	46	46 (34.2)	χ^2^ = 0.000, P = 0.988,	OR = 1.005, 95 % CI 0.559–1.804
	Yes	27	27 (34.1)		

## Discussion

Chlamydia has been identified as an accelerator in the transmission and acquisition of HIV [19]. Most women and about half of men with chlamydia do not experience symptoms [20]. Since symptoms may not be present, the only way to know if a person who may be at risk, is infected with chlamydia is to be tested. In the present study, 243 urine samples were collected and tested by real time PCR. The overall prevalence of chlamydia in the present study is higher compared to those described in other parts of South Africa even when similar population (HIV positive) was used [21]. The prevalence of *C. trachomatis* was significantly higher in female patients (39.2 %) than in male patients (15.5 %) (P = 0.001). This could be due to chlamydia being asymptomatic in most cases particularly in females who therefore do not test and get treated. Similar results were obtained in a study on chlamydia prevalence in the general population in Netherlands were the prevalence was high in females than males [22]. Another study in Estonia found a high prevalence of 48.0 % in females than 32.0 % in males [23].

In Tanzania a study on the prevalence of HIV and *C. trachomatis* infection in 15–19-year olds in rural Tanzania found a prevalence of 2.4 % in females and 1.0 % in males. However other studies have shown a non-significant difference between males and females [24]. For example a study in the United States on gender and age disparities in the prevalence of chlamydia infection among sexually active adults found that the observed difference in CT prevalence between females and males was not statistically significant (1.6 vs. 1.7 %) with the P value of 0.8 [25]. In high risk groups, chlamydial infection was found to be correlating with various socio-demographic characteristics, such as low education or being single. These associations are far less significant in the general population and tend to incorrectly classify many women at high risk of chlamydial infection, and therefore reduction in screening effectiveness [26].

In the present study, we found that widowed and divorced individuals had more CT infections compared to unmarried people. This situation is contrary to those described by other authors who found higher prevalence among single people compared to married people. A study by Kolvstad and his colleagues showed a higher prevalence of CT among single women (6.6 %) compared to married women (0 %) [27]. However, a study in Jos, Nigeria among gynecologic clinic attendees found a higher prevalence of chlamydia in married women (38.41 %) compared to single women (17.07 %) and the divorced (0.61 %) [28]. In our study, a higher prevalence was observed among widowed patients (46.7 %) although the difference was not statistically significant (P = 0.262). These differences could be due to different populations dynamics in these countries, as well as the type of study population used. In another study, younger age, living alone or with persons other than partners/family members, engaging in unprotected vaginal intercourse (UVI) and having other STIs seemed to be associated with higher risk of CT infection [29]. Similarly, being divorced/widowed and working in middle/low-level venues were identified as additional risk factors for other STI’s such as gonorrhea.

Younger patient’s age has been associated with greater risk of chlamydia [30], because the cervix of teenage girls and young women is not fully matured and is probably more susceptible to infection, they are at particularly hi particularly high risk for infection if they are sexually active. Previous studies have demonstrated that *C. trachomatis* infection is most common in people aged less than 25 years, with rates decreasing thereafter [31, 32]. In the present study, the prevalence was found to be high among people aged between 25–45 years (35.6 %). Similar results have been reported by previous studies in the African continent. For example, a high prevalence of 43.9 % was reported in people of between 26–30 years in a study on the prevalence of chlamydia in patients attending gynecological clinics in South Eastern Nigeria [33]. A previous study in Mpumalanga Province found a high prevalence of (47.9 %) in male and (52.8 %) in female miners particularly those who were aged between 46–50 years old [34].

In the present study, out of 235 HIV positive patients a 31.9 % prevalence was found. There were only 8 HIV negative patients, among whom 3 (37.5 %) were chlamydia positive. This could mean that the prevalence of chlamydia is not always associated with the reported HIV status. Similar results were obtained in the Mopani District South Africa in a study on prevalence of chlamydia and gonorrhea among HIV infected women in rural Mopani District [35]. The use of ARV also appeared to have effects on the infection rate because the percentage was high in HIV patients who were not taking ARV’s (38.1 %) than those who were taking it (28.5 %), therefore antiretroviral therapy could be helpful in reducing the risks and sequelae of chlamydia infection.

Socio-economical status of study participants has often been cited as risk factor for the occurrence of several diseases. In our study, we found that the prevalence of CT was higher among people who admitted to having an income of less than 1000 Rands per month (35.4 %) although the difference was not significant (P = 0.425). This correlates with a study that was conducted in the United States by Klovstad et al. [27], where the percentage in people who were unemployed was higher compared to those who were employed but also the difference was not significant. In our study a prevalence of 35.9 % was obtained among people who only studied until secondary school although the difference was not significant with other educational levels. Beydoun and colleagues [25] reported a higher prevalence in people who studied until high school only (4.8 %) compared to those who studied further until tertiary (0.8 %) and the difference was significant (P = 0.004).

People who had their first sexual intercourse at an early age were at higher risk of infection. We found the highest prevalence of chlamydia among people who started being sexually active between 12–15 years (43.8 %) although this difference was not statistically significant (P = 0.153). A study in Belgium focusing on chlamydial opportunistic screening in general practice found a significant difference (P = 0.008) between people who had their first sexual intercourse at a younger age [26]. Similarly, Mawak et al. [28], found high prevalence among people who had their first sexual intercourse at ages between 15–19 years, but the difference was also not significant (P = 0.147).

People with multiple sexual partners in the previous year appeared to have more infection [27]. Although number of sexual partners has been found to be statistically associated with *C. trachomatis* infection in other studies, Mawak and his colleagues reported that 37.2 % of people with multiple sexual partners tested positive for chlamydia but the difference was not statistically significant [28]. In the present study a high prevalence was found among people who indicated that they had had only one sexual partner in the previous year (38.6 %). Verhoeven in 2012 reported a prevalence of 17.9 % in people who had more than 6 sexual partners in the previous year with the difference being statistically significant P = 0.000 [26]. In a population based survey in Estonia on the prevalence of chlamydia, the number of sexual partners in the past 12 months was the strongest predictor of the infection because participants with 2 or more partners had the highest prevalence (11.8 %) when compared to those with only one sex partner (3.8 %) and the difference was statistically significant (P = 0.002) [23].

Previous epidemiological studies reported that unprotected sex was one of the major causes of sexually transmitted infections and a modifiable risk factor. In 2012 Dubbink and colleagues also reported a higher prevalence (40 %) in people who did not use condoms in the Mopani District South Africa [35]. In our study, a high prevalence was found in people who indicated that they use condoms sometimes (44.7 %) compared to those who said they use condoms always (33 %), however the difference was not statistically significant (P = 0.262). A prevalence of (4.41 %) in people who never use condoms was found in Cameroon [36]. Klovstad and colleagues [27] also reported a higher prevalence (10.1 %) among sexually active men who have unprotected sex.

In the present study we also looked at the potential associations between CT infections and other clinical characteristics that the patients had experienced such as genital warts, sores on genitals, sores on lips, rash on genitals, mouth rash and history of other STD’s, associations with the infection were noted. A non-significant incidence of 7.5 % in people with genital warts has been previously reported [27]. Presence or history of other STD’s has also been identified as a risk factor for CT [29]. Similar outcomes were obtained by Ngadjio and colleagues [36] who found that people who had history of other STI’s had 6.19 % prevalence compared to 2.43 % among people who did not have other STI’s [34]. A previous study in Mpumalanga, South Africa on measuring the impact of HIV and STI’s in a community in a coal mining town, found a prevalence of 19 % in people who had a history of other STI’s. History of other STI’s was also reported as one of the risk factors for the chlamydia infection with 41.46 % which was statistically significant [28]. Other conditions such as pregnancy, history of abortion, miscarriage also appear to be in relation to the chlamydia infection. Mawak and colleagues reported on history of abortion (26.22 %) as a result of this infection [28].

## Conclusions

In conclusion, the prevalence of Chlamydia was determined in HIV and AIDS patients in the Vhembe District. The prevalence was higher among young people and this emphasizes the need to enhance STI control among all young people. The use of ARV appeared to be beneficial in reducing the prevalence of chlamydia among HIV positive patients. However further studies are needed in the general population both HIV positive and HIV negative persons to further determine the impact of these infections in the community.

## Abbreviations

STI: sexually transmitted infections,
ARV: antiretroviral,
CT: *Chlamydia trachomatis*
